# Comparison of the gut microbiota and metabolites between Diannan small ear pigs and Diqing Tibetan pigs

**DOI:** 10.3389/fmicb.2023.1197981

**Published:** 2023-07-06

**Authors:** Xuancheng Guan, Junhong Zhu, Lanlan Yi, Haichao Sun, Minghua Yang, Ying Huang, Hongbin Pan, Hongjiang Wei, Hongye Zhao, Yanguang Zhao, Sumei Zhao

**Affiliations:** ^1^Yunnan Key Laboratory of Animal Nutrition and Feed Science, Yunnan Agricultural University, Kunming, China; ^2^Key Laboratory for Porcine Gene Editing and Xenotransplantation in Yunnan Province, Kunming, China; ^3^Shanghai Laboratory Animal Research Center, Shanghai, China

**Keywords:** Diannan small ear pigs, Diqing Tibetan pigs, gut microbiota, metabolites, 16S rRNA, LC-MS

## Abstract

**Objective:**

Host genetics and environment participate in the shaping of gut microbiota. Diannan small ear pigs and Diqing Tibetan pigs are excellent native pig breeds in China and live in different environments. However, the gut microbiota of Diannan small ear pigs and Diqing Tibetan pigs were still rarely understood. Therefore, this study aimed to analyze the composition characteristics of gut microbiota and metabolites in Diannan small ear pigs and Diqing Tibetan pigs.

**Methods:**

Fresh feces of 6 pigs were randomly collected from 20 4-month-old Diannan small ear pigs (DA group) and 20 4-month-old Diqing Tibetan pigs (TA group) for high-throughput 16S rRNA sequencing and liquid chromatography-mass spectrometry (LC-MS) non-targeted metabolome analysis.

**Results:**

The results revealed that Firmicutes and Bacteroidetes were the dominant phyla in the two groups. Chao1 and ACE indices differed substantially between DA and TA groups. Compared with the DA group, the relative abundance of Prevotellaceae, and *Ruminococcus* was significantly enriched in the TA group, while the relative abundance of Lachnospiraceae, *Actinomyces*, and *Butyricicoccus* was significantly reduced. Cholecalciferol, 5-dehydroepisterol, stigmasterol, adrenic acid, and docosahexaenoic acid were significantly enriched in DA group, which was involved in the steroid biosynthesis and biosynthesis of unsaturated fatty acids. 3-phenylpropanoic acid, L-tyrosine, phedrine, rhizoctin B, and rhizoctin D were significantly enriched in TA group, which was involved in the phenylalanine metabolism and phosphonate and phosphinate metabolism.

**Conclusion:**

We found that significant differences in gut microbiota composition and metabolite between Diannan small ear pigs and Diqing Tibetan pigs, which provide a theoretical basis for exploring the relationship between gut microbiota and pig breeds.

## Introduction

Pigs are an important livestock species, and pork is essential in human daily meat protein intake (Xiao et al., 2018). Nowadays, pork represents more than 30% of the worldwide meat market, second only to chicken (Oh and See, 2012). The National Commission for Livestock and Poultry Genetic Resources of China has recognized 48 local pig breeds (Fang et al., 2005; Peng et al., 2022). Yunnan province is situated in China’s southwestern region. Its unusual geographical position, complicated topography, unique climate, and various national farming traditions have produced abundant native pig resources, of which the Diannan small ear pigs and the Diqing Tibetan pigs are noteworthy examples (Shang et al., 2020; Wu et al., 2020).

**Figure 1:**
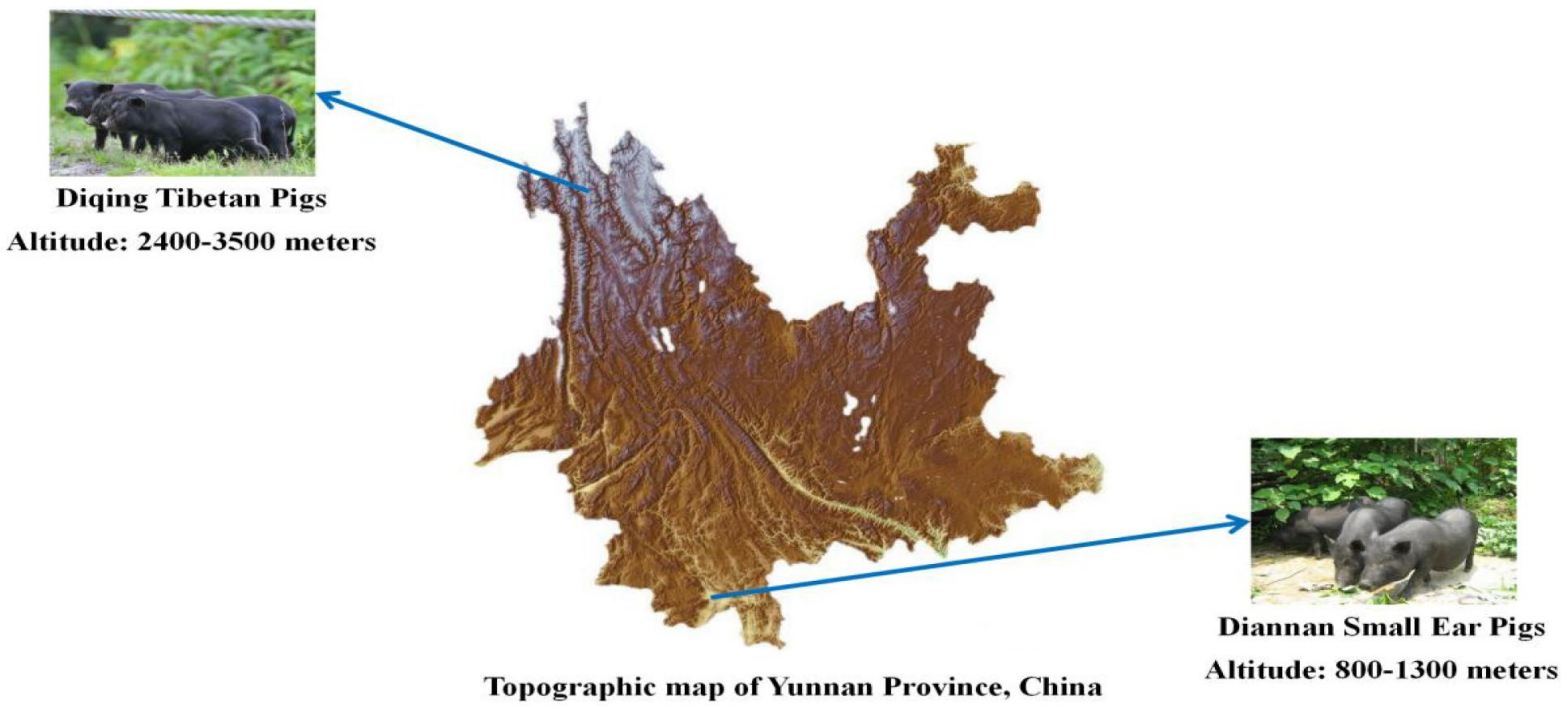
Geographical distribution of Diannan small ear pigs and Diqing Tibetan pigs.

Diannan small ear pigs live at the low altitude area in the south of Yunnan province, with an altitude of 800–1,300 m, belonging to the subtropical climate (Yonggang, 2010). Compared to European pig breeds such as Duroc, Landrace, and Yorkshire pigs, the Diannan small ear pigs has exceptional resistance to coarse food and excellent meat quality (Wang et al., 2016). Diqing Tibetan pigs live at an average elevation of approximately 3,500 m on the Yunnan Plateau (Li et al., 2013). Diqing Tibetan pigs have developed complex phenotypic and physiological adaptations to high-altitude hypoxia compared with lowland pigs (Zhang J. et al., 2015; Zhang B. et al., 2015). In summary, Diannan small ear pigs and Diqing Tibetan pigs have abundant phenotypic traits and superior commercial traits, adaptability to different environments (Figure 1), so it is regarded as valuable genetic resource that deserves to be efficiently utilized for scientific conservation and commercial exploitation.

A dense, dynamic, and highly complex microbial community inhabits the animal gastrointestinal tract (Yang et al., 2016). Intestinal microorganisms are essential and mobile animal metabolic organs that maintain body homeostasis (LeBlanc et al., 2013). Intestinal microorganisms may be influenced by the host heredity and environmental (Ley et al., 2006; Sutherland et al., 2006; Miao et al., 2009; Guo et al., 2011; Wu et al., 2013). Presently, comparative studies on the intestinal microorganisms of Diannan small ear pigs and Banna small pig inbred lines, Tibetan Pigs and Duroc pigs, Jinhua Pigs, and Landrace pigs have been conducted by the predecessors (Xiao et al., 2018; Wei et al., 2020; Yang et al., 2022). However, there are few reports on the comparative study of intestinal microorganisms between the Diannan small ear pigs and the Diqing Tibetan Pigs. Exploring the effect of host genetics on gut microbiota and metabolites can help reveal the metabolic interactions between microorganisms and hosts *in vivo*, which is important for understanding the gut health of hosts and improving the productivity of pig industry.

This study aims to analyze the composition characteristics of gut microbiota and metabolites in Diannan small ear pigs and Diqing Tibetan pigs and investigate the relationship between pig germplasm features and gut microbiota and metabolites. To provide a theoretical basis for exploring the relationship between gut microbiota and pig breeds.

## Materials and methods

### Animals and sample collection

All animal procedures were performed according to the Guide for Animal Care and Use of Laboratory Animals in the Institutional Animal Care and Use Committee of Yunnan Agricultural University. The Department Animal Ethics Committee approved the experimental protocol of the Yunnan Agricultural University. Twenty Diannan small ear pigs (4 months of age, 39 ± 7.35 kg) and twenty Diqing Tibetan pigs (4 months of age, 38 ± 6.96 kg) raised by the pig-breeding institute of the Yunnan Academy of Animal Husbandry and Veterinary Sciences were selected. Each pig is housed in a pig house with the same environment and raised for 30 days using a standardized feeding scheme (Supplementary Table S1). Then, randomly select 6 Diannan small ear sows (DA group) and 6 Diqing Tibetan sows (TA group), collect fresh feces through rectal swabs, quickly freeze them in liquid nitrogen, and store them at −80°C.

### Genomic DNA extraction and 16S rRNA gene sequencing

The fecal DNA extraction kit was utilized to extract the samples’ total DNA (Beijing tiangen biochemical technology co., LTD.). Using agarose gel electrophoresis, the purity and concentration of DNA were assessed, and an adequate amount of sample DNA was deposited in a centrifuge tube. The sample was diluted with sterile water to 1 ng/μL. The diluted genomic DNA was utilized as the template for PCR expansion, with the V3-V4 region of the 16S rRNA gene being amplified. The extended primers were 338F (5′-ACTCCTACGGGAGGCAGCA-3′) and 806R (5′-GGACTACHVGGGTWTCTAAT-3′). The Barcode-specific primers were used. PCR products were identified using 2% agarose gel electrophoresis. According to the concentration of the PCR product, the same amount of samples was combined with the PCR product. After carefully combining the samples, the PCR product was purified by 2% 1 × TAE gel electrophoresis, and the target band was cut and recovered (Thermo Scientific company GeneJET adhesive recovery kit). Thermofisher’s Ion Plus Fragment Library Kit 48 RXNS was used to construct the Library. After the library was validated by Qubit quantitative and library testing, Thermofisher’s Ion S5^™^XL was implemented for computer sequencing.

### Sequence processing and data analysis

Cutadapt (Version 1.9.1^1^) was utilized to remove low-quality portions of reads, which were then matched to the species annotation database to identify chimeric sequences (Aßhauer et al., 2015). Chimera sequences were eliminated in order to acquire the final reliable data (Clean Reads) (Uparse version 7.0.1001^2^) (Haas et al., 2011), and by default, the sequence was clustered as Operational Taxonomical Units (OTUs) with 97% identity. Mothur technique and SSUrRNA database of SILVA132 (threshold set to 0.8) were utilized to perform species annotation on OTUs sequences (Edgar, 2013). Rapid multiple sequence alignment utilizing MUSCLE software (Version 3.8.31) to determine system occurrence relationships for all OTUs sequences (Quast and Pruesse, 2013). QIIME software was used to examine the samples for Alpha diversity and Beta diversity (Version 1.9.1). By calculating the unifrac distance between OTUs in samples (Lozupone and Knight, 2005; Lozupone et al., 2011), unweighted group average clustering analysis (UPGMA) was created. The screening value of the linear discrimination criterion (LDA Score) was 2 using the LEfSe program for inter-group differential species analysis (Segata et al., 2011). Tax4Fun may be used to forecast the function of all samples (Qin et al., 2012).

### LC-MS analysis of fecal metabolites

Collect the liquid sample for analysis 100 μL (0.1 mg fecal tissue liquid nitrogen grinding) put in centrifuge tube, add in 400 including 80% of methanol aqueous solution (4 times the volume of methanol), vortex oscillation, in −20°C stand for 60 min, 14,000 g, 4°C centrifugal 20 min, take a certain amount of supernatant put in 1.5 mL centrifuge tube, vacuum freeze-drying, the residue with 100 μL complex solvents to dissolve, vortex oscillation, 14,000 g, 4°C centrifugal 15 min, take that into the supernatant fluid sample LC-MS analysis.

Chromatographic conditions: positive model: mobile phase A: 0.1% formic acid, 95% acetonitrile, 10 mM ammonium acetate; Mobile phase B: 0.1% formic acid, 50% acetonitrile, 10 mM ammonium acetate. Negative mode: mobile phase A: 95% acetonitrile, 10 mM ammonium acetate, pH 9.0; Mobile phase B: 50% acetonitrile,10 mM ammonium acetate, pH 9.0. Column temperature: 40°C; Flow rate: 0.3 mL/min. Mass spectrometry conditions: the scanning range of 100–1,500 m/z was used. The ESI Settings are as follows: Spray Voltage: 3.2kv; Sheath gas flow rate: 35 arb; Aux Gas flow rate: 10 arb; Temperature: 320°C. Polarity: positive; Negative; MS/MS level 2 scans are data-dependent scans.

Load the output data (raw) file into compound discoverer (CD) search software for straightforward filtering of retention duration, mass charge ratio, and additional characteristics. Several samples were then aligned according to a retention time deviation of 0.2 min and a mass deviation of 5 parts per million to improve the accuracy of the identification. The peak was recovered based on the specified mass deviation of 5 ppm, signal strength deviation of 30%, signal-to-noise ratio of 3, minimum signal strength of 100,000, and other data. The peak area was quantified, the target ions were integrated, the molecular formula was predicted and compared with the mzCloud database, the background ions were removed with blank samples, the quantitative results were normalized with quality control (QC) samples, and the identification and quantitative results of the data were obtained.

### Correlation analysis

On the basis of the correlation coefficient (r) of the different microbiota and metabolites, SPSS 22.0 was used to conduct a Pearson correlation analysis, and cytoscape (version 3.5.1) was utilized to create a correlation heat map.

## Results

### Gut microbiota: analysis and function prediction

**Figure 2:**
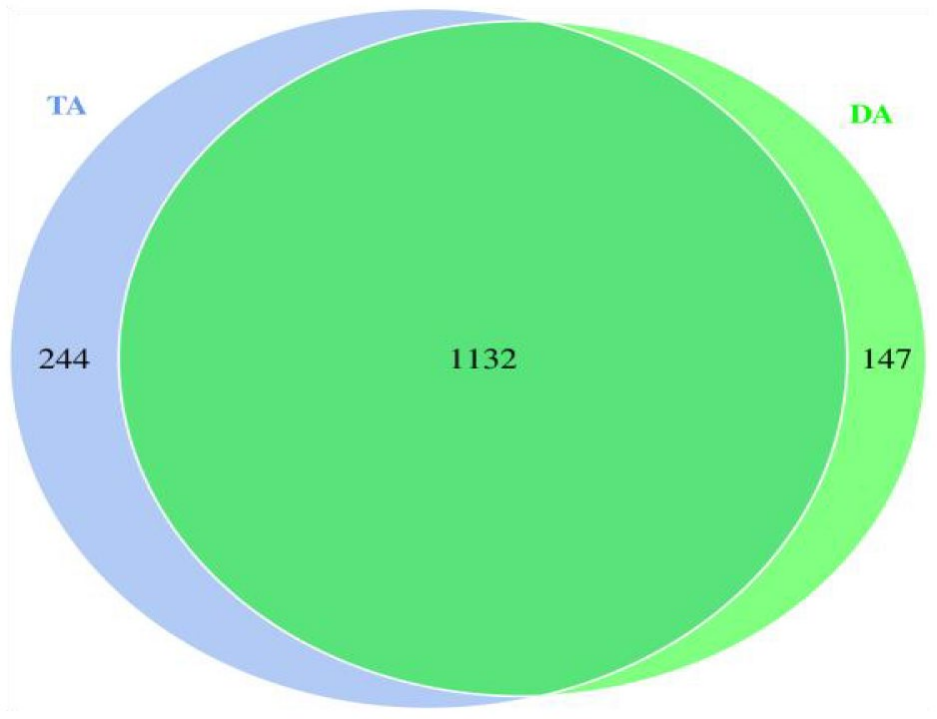
Venn diagram of OTU.

Comparing the OTUs of the two groups determined that the number of identical OTUs in the DA and TA groups was 1,132, while the number of special OTUs in the DA and TA groups was 147 and 244, respectively (Figure 2).

**Figure 3:**
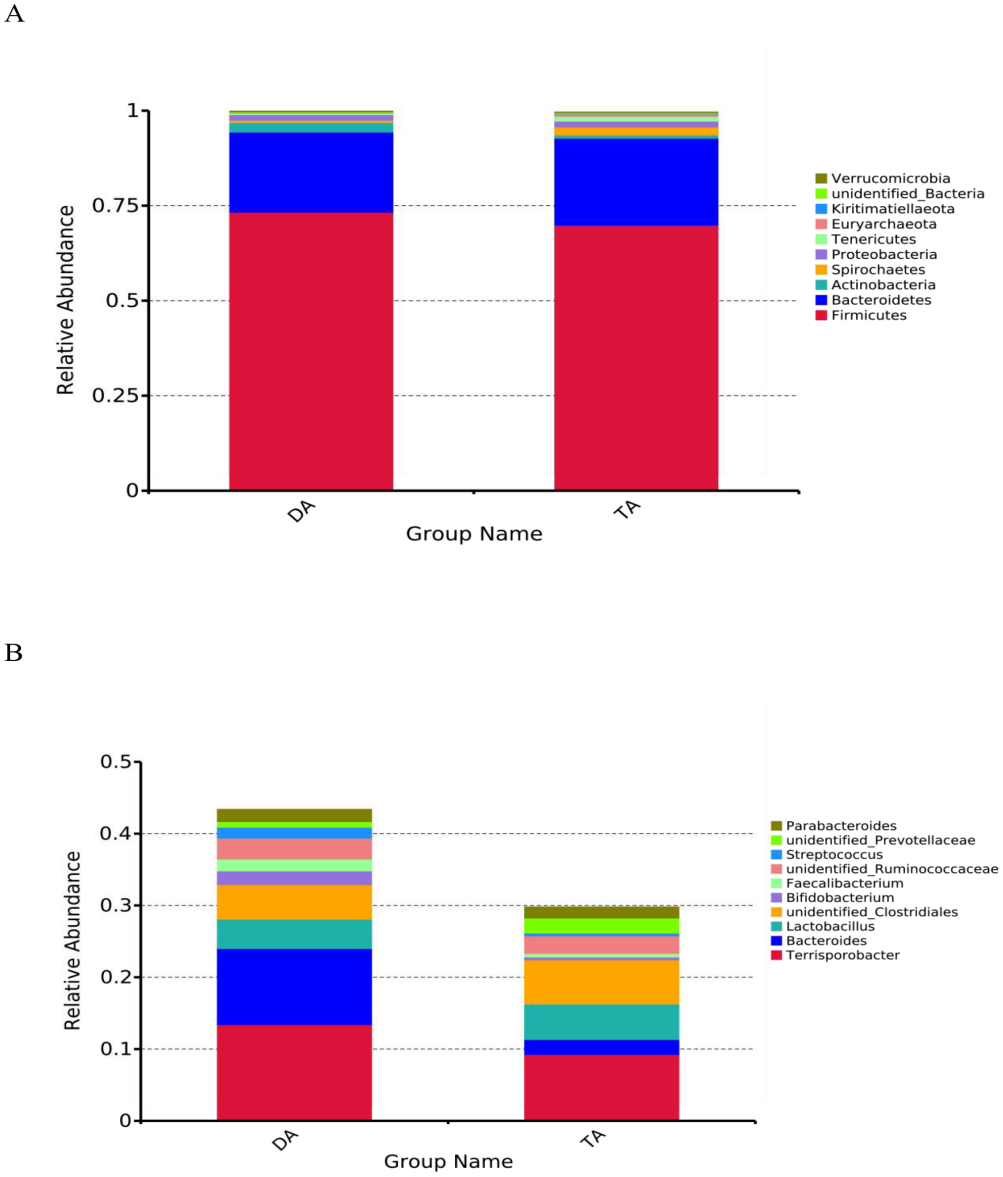
The histogram of relative abundance for intestinal microbiota in Diannan small ear pigs and Diqing Tibetan pigs (select the top 10 microbes at each level). **(A)** Phylum level. **(B)** Genus level.

At the phylum level, the top 10 microorganisms in the relative abundance of the DA group and TA group are Firmicutes, Bacteroidetes, Actinobacteria, Spirochaetes, Proteobacteria, Tenericutes, Euryarchaeota, Kiritimatiellaeota, unidentified_Bacteria, Verrucomicrobia, among which Firmicutes and Bacteroidetes were the major phylum of bacteria in both the DA and TA groups, accounting for more than 90% of total microbiota in both groups (Figure 3A). The relative abundance of Firmicutes and Actinobacteria in the DA group was higher than that in the TA Group. The relative abundance of Bacteroidetes, Spirochaetes, Proteobacteria, and Tenericutes in the TA Group was higher than that in the DA group.

At the genus level, the top ten microorganisms in the relative abundance of the DA and TA groups are *Terrisporobacter*, *Bacteroides*, *Lactobacillus*, *unidentified_Clostridiales*, *Bifidobacterium*, *Faecalibacterium*, *unidentified_Ruminococcaceae*, *Streptococcus*, *unidentified_Prevotellaceae*, *Parabacteroides* (Figure 3B). The relative abundance of *Terrisporobacter, Bacteroides, Bifidobacterium, Faecalibacterium,* and *Streptococcus* in the DA group was higher than that in the TA group. The relative abundance of *Lactobacillus, unidentified_Clostridiales, unidentified_Ruminococcaceae, unidentified_Prevotellaceae,* and *Parabacteroides* in the TA group was higher than that in the DA group.

**Figure 4:**
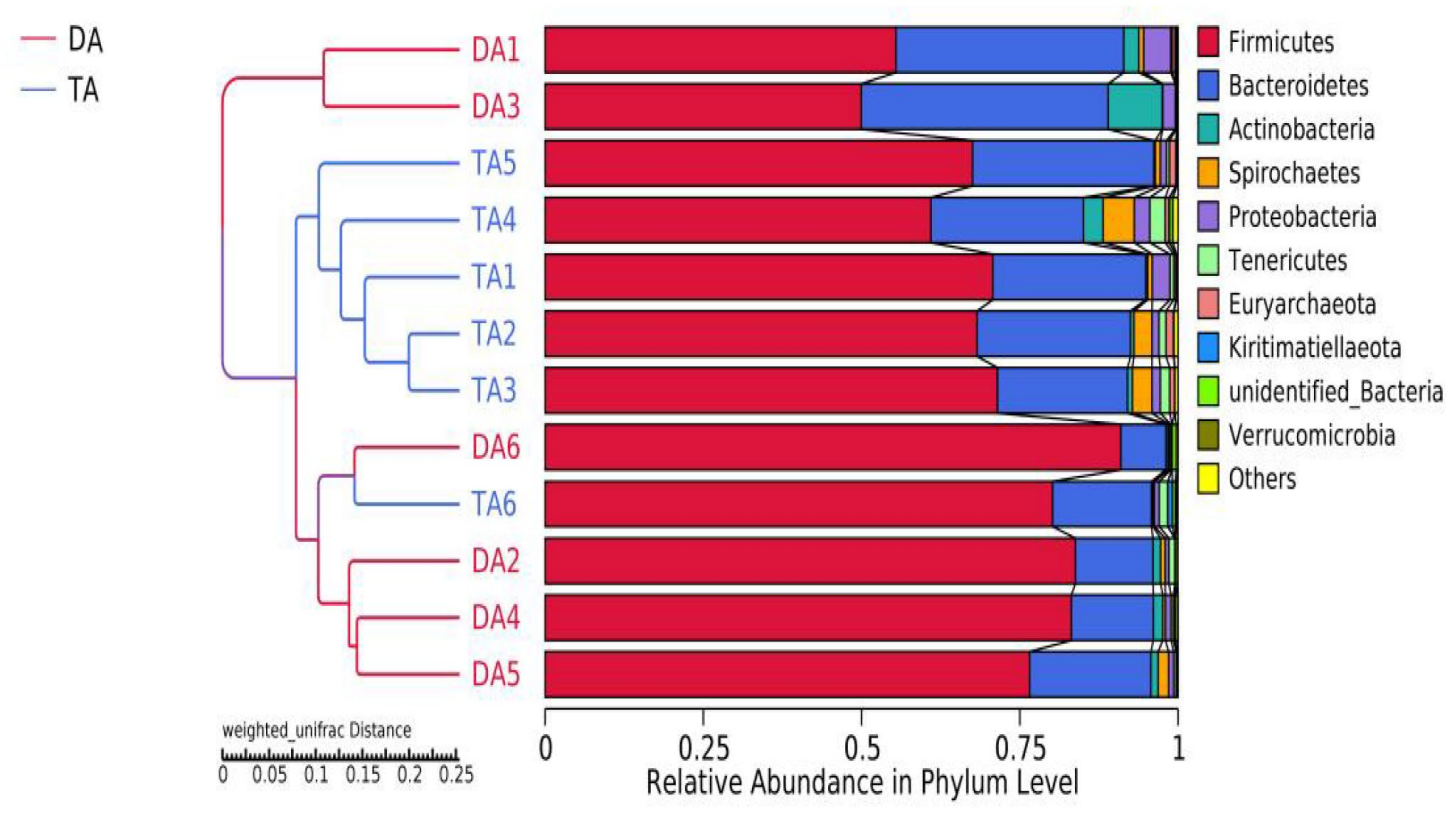
The beta diversity results of different grouped samples-UPGMA clustering tree.

After 97% identity clustering, the DA group obtained an average of 741 OTUs, while the TA group obtained an average of 847 OTUs. OTUs are then further annotated to calculate and compare the Alpha diversity between samples (Table 1). The Chao1 and ACE indices of the TA group were significantly higher than those of the DA group (*p* < 0.05), indicating that the TA group had a greater diversity of intestinal microbial than the DA group. UPGMA with weighted mean revealed that DA and TA samples are clustered in distinct branches, indicating that the microbiota structure of these two groups is distinct (Figure 4).

**Table 1:** The Alpha diversity results of different grouped samples.

Sample name	DA	TA
Observed species	741.00 ± 83.62^a^	846.50 ± 68.96^b^
Shannon index	6.60 ± 0.89	6.97 ± 0.68
Simpson index	0.96 ± 0.36	0.97 ± 0.25
Chao1 index	804.64 ± 76.84^a^	900.88 ± 60.72^b^
ACE index	806.44 ± 76.92^a^	901.46 ± 59.65^b^
goods coverage	0.99 ± 0.00	0.99 ± 0.00
PD whole tree	57.98 ± 5.85	64.91 ± 6.49

In the same row, values with different letter superscripts mean significant difference (*p* < 0.05), while with no letter superscripts mean no significant difference (*p* > 0.05).

**Figure 5:**
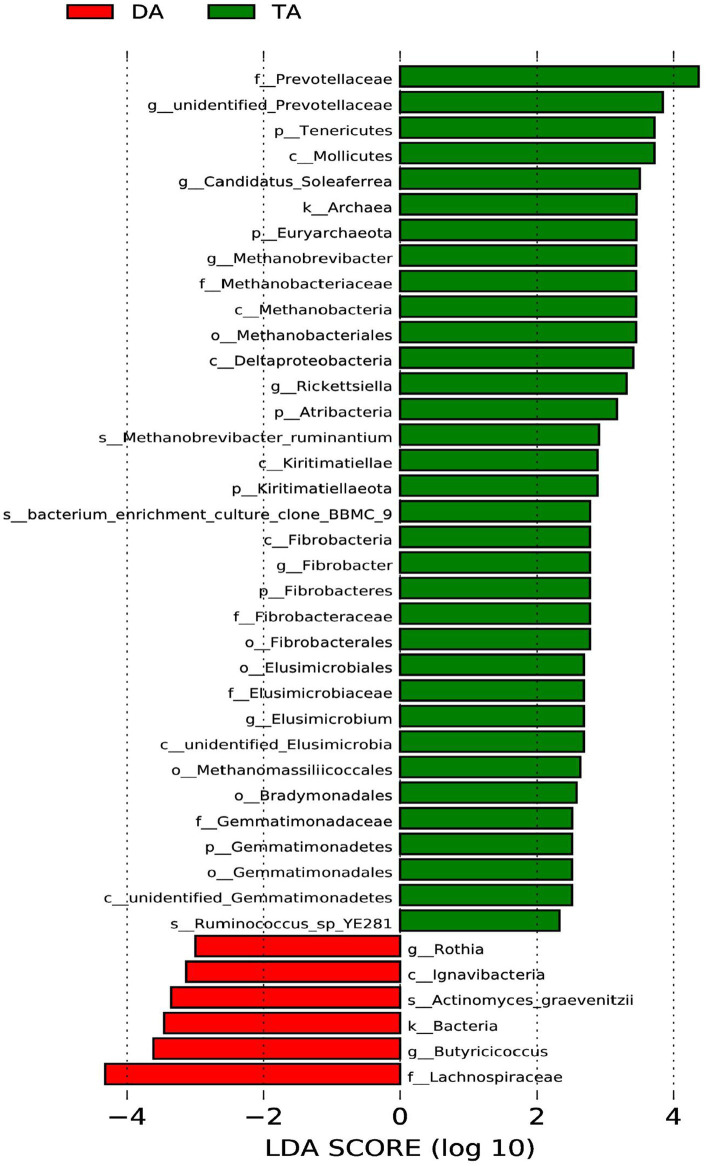
Analysis of LEfSe multi-level species difference discriminant.

LEfSe multistage discriminant analysis showed that there were 40 biomarkers (LDA score ≥ 2) at different classification levels in the intestinal microbial of pigs in the DA and TA groups (Figure 5). At the phylum level, the relative abundance of Tenericutes, Fibrobacteres, Atribacteria, Kiritimatiellaeota, Gemmatimonadetes, and Euryarchaeota in the TA group was significantly higher than that in the DA group (*p* < 0.05); At the class level, the abundance of Mollicutes, Methanobacteria, Deltaproteobacteria, Kiritimatiellae, unidentified_Elusimicrobia, unidentified_Gemmatimonadetes in the TA group was significantly higher than that in DA group (*p* < 0.05). While the abundance of Ignavibacteria in the DA group was significantly higher than that in the TA group (*p* < 0.05); At the order level, the abundance of Methanobacteriales, Fibrobacterales, Elusimicrobiales, Methanomassiliicoccales, Bradymonadales, Gemmatimonadales in the TA group was significantly higher than that in DA group (*p* < 0.05); At the family level, the abundance of Prevotellaceae, Methanobacteriaceae, Fibrobacteraceae, Elusimicrobiaceae, Gemmatimonadaceae in the TA group was significantly higher than that in DA group. While the abundance of Lachnospiraceae in the DA group was significantly higher than that in the TA group (*p* < 0.05). At the genus level, the abundance of *unidentified_Prevotellaceae*, *Candidatus_Soleaferrea*, *Methanobrevibacter*, *Rickettsiella*, *Fibrobacter*, *Elusimicrobium* in the TA group was significantly higher than that in the DA group (*p* < 0.05). While the abundance of *Rothia* and *Butyricicoccus* in the DA group was significantly higher than that in the TA group (*p* < 0.05); At the species level, the *abundance of Ruminococcus_sp_YE281*, *bacterium_enrichment_culture_clone_BBMC_9*, *Methanobrevibacter_ruminantium* in the TA group was significantly higher than that in DA group. In contrast, the abundance of *Actinomyces_graevenitzii* in the DA group was significantly higher than that in the TA group (*p* < 0.05).

**Figure 6:**
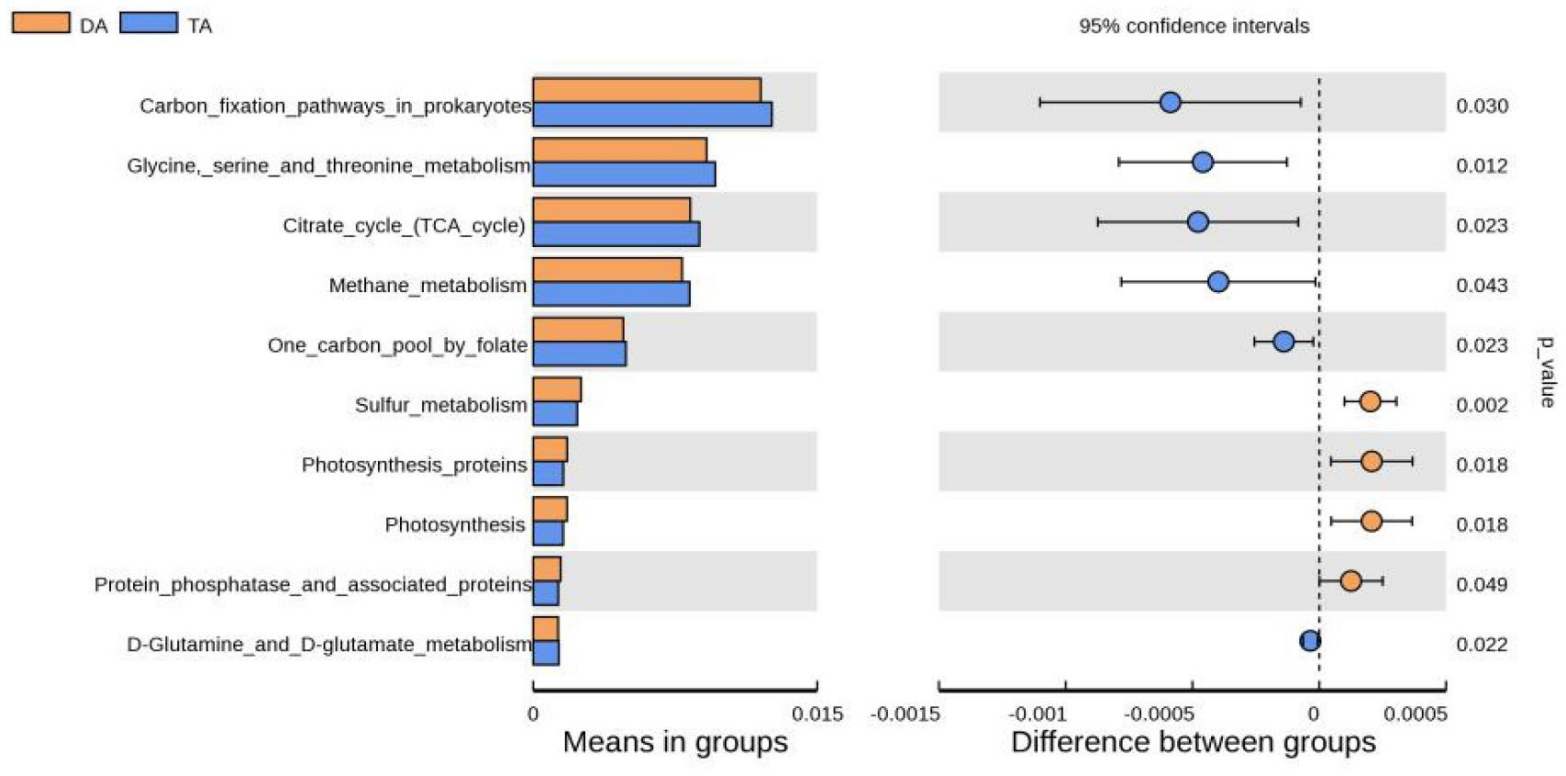
The function annotation abundance map of KEGG level 3.

Tax4Fun software was used for functional prediction analysis (Figure 6). The result showed that at the KEGG level 3, DA and TA groups were mainly involved in carbon fixation pathways in prokaryotes, glycine serine and threonine metabolism, citrate cycle (TCA cycle), methane metabolism, one carbon pooly folate, sulfur metabolism, protein phosphatase and associated proteins, D-Glutamine and D-glutamate metabolism. Among them, carbon fixation pathways in prokaryotes, glycine serine and threonine metabolism, TCA cycle, methane metabolism, one carbon pool by folate, D-Glutamine and D-glutamate metabolism was significantly enriched in TA group, while sulfur metabolism, protein phosphatase and associated proteins was significantly enriched in DA Group.

### Metabolome data analysis

**Figure 7:**
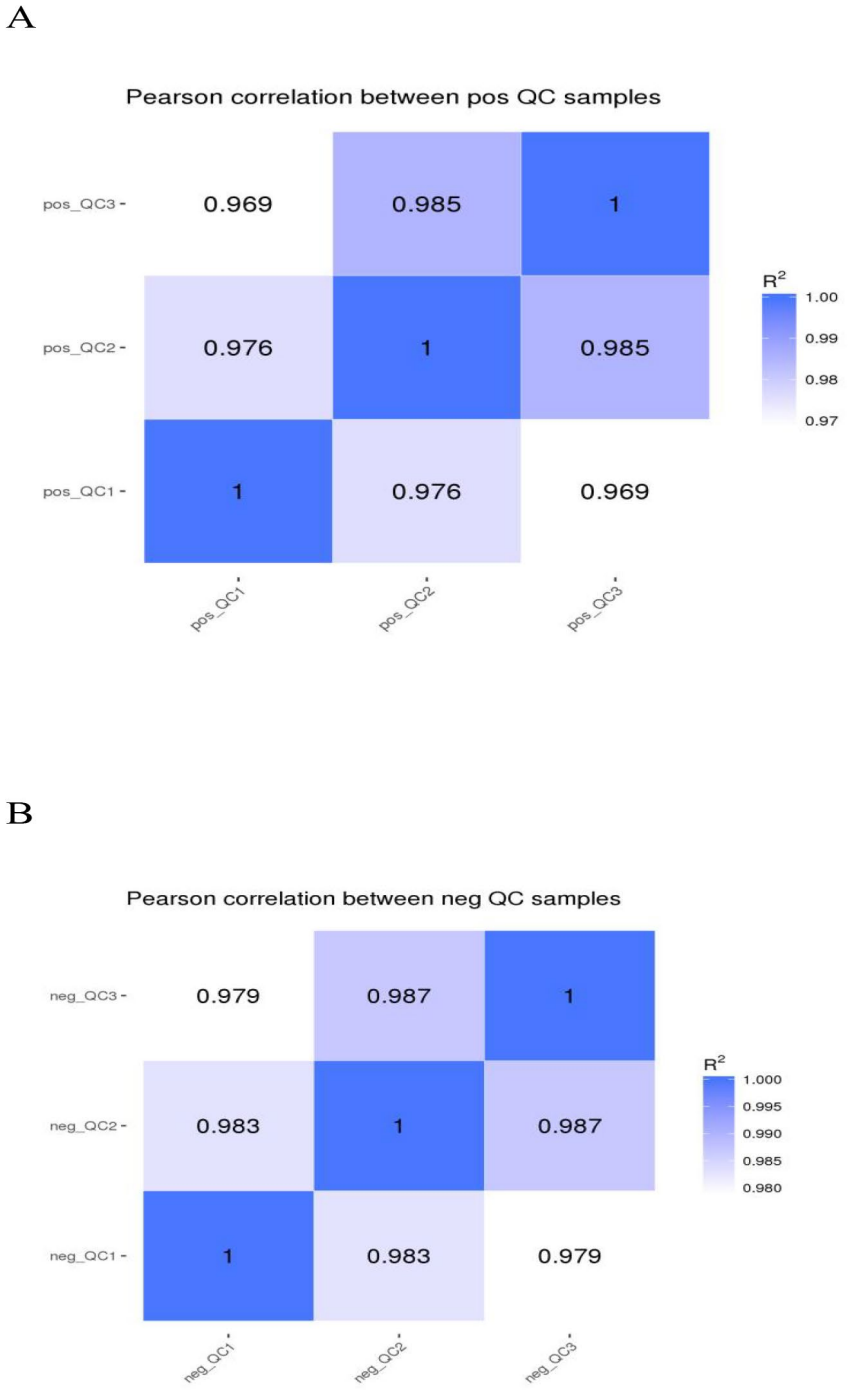
QC quality control. **(A)** Positive ion mode. **(B)** Negative ion mode.

**Figure 8:**
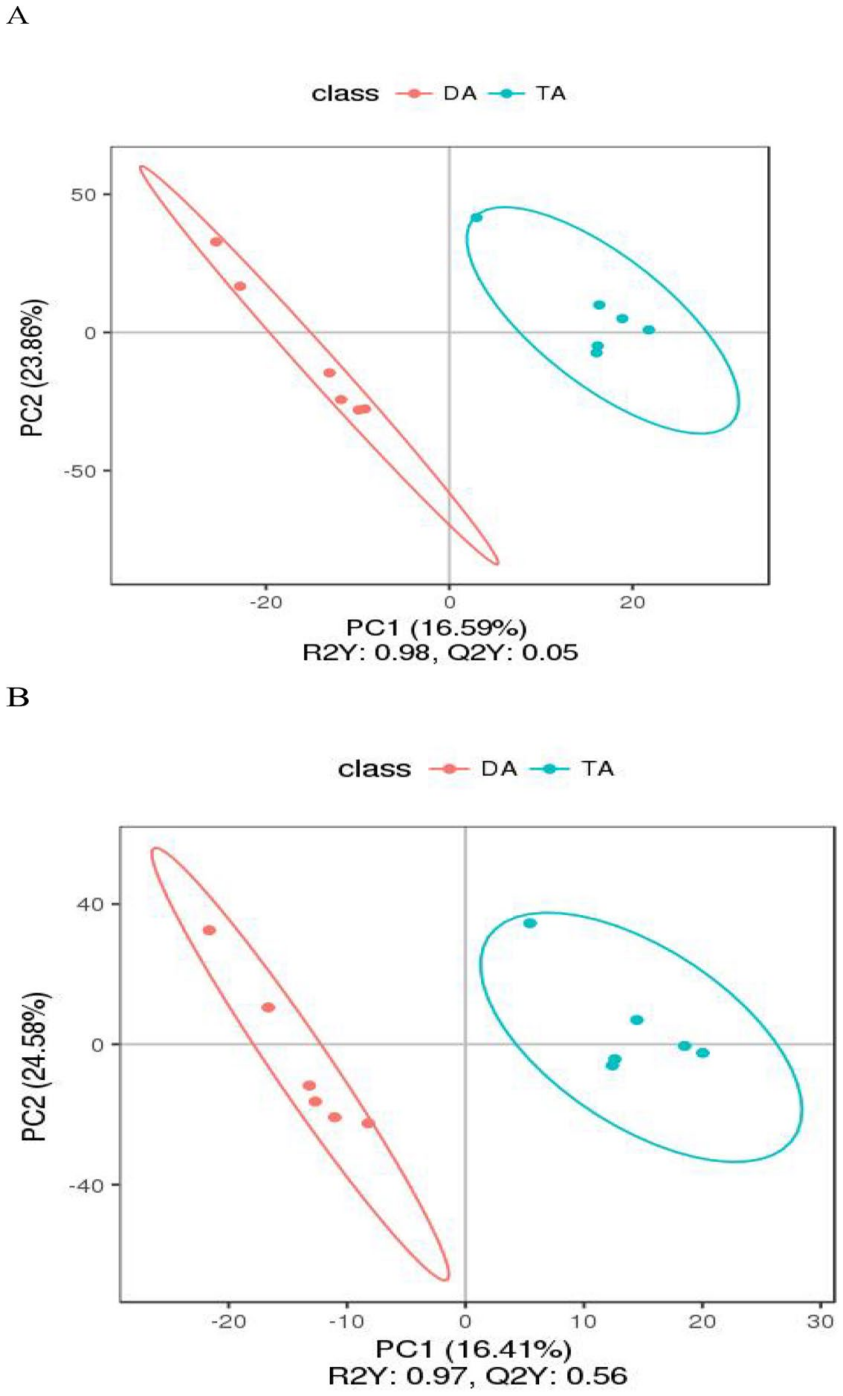
PLS-DA chart. **(A)** Positive ion mode. **(B)** Negative ion mode.

In the positive and negative ion modes, the correlation of QC samples and *R*^2^ values are close to 1 (Figure 7). The contributions of PC1 and PC2 were 16.59 and 23.86% in the positive ion mode and 22.00 and 16.28% in the negative ion mode, respectively. Both groups were effectively distinguished in the positive and negative ion modes (Figure 8). 2,505 and 1,848 metabolites were detected in fecal samples of DA group and TA group, respectively. Among the top 10 metabolites with relative expression, oleamide is the metabolite with the highest relative expression in the positive and negative ion mode of the two groups (Supplementary Tables S2–S5).

**Figure 9:**
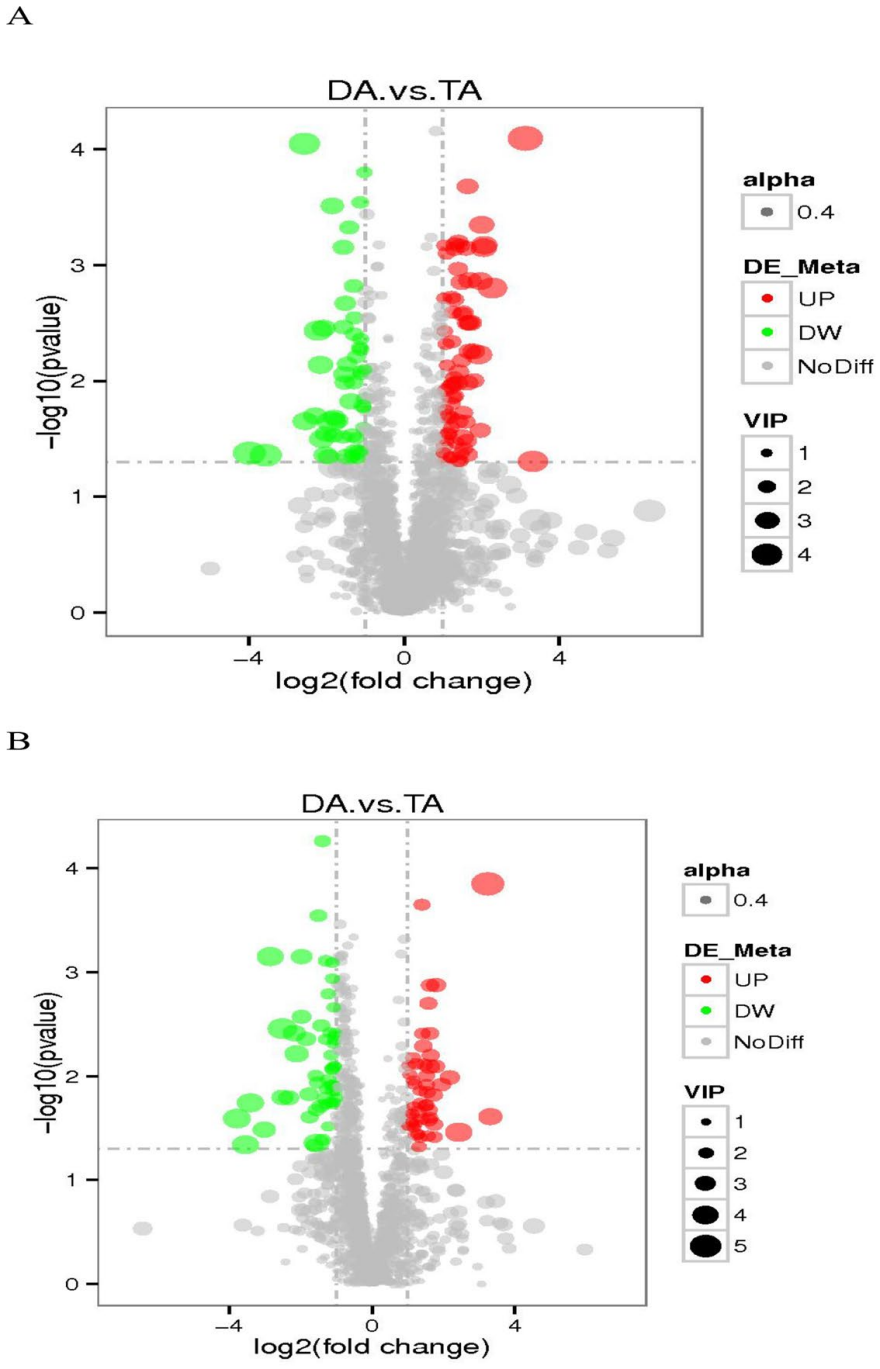
The volcano map of differential metabolites. **(A)** Positive ion mode. **(B)** Negative ion mode.

The threshold was set as VIP > 1.0, Fold Change (FC) > 2.0 and FC < 0.5, and *p* value < 0.05, and the differentially expressed metabolites were screened out (Figure 9). In the positive ion mode, there were 124 metabolites with significant differences between the DA and TA groups, among which 73 were significantly up-regulated and 51 were significantly down-regulated; In the negative ion mode, there were 105 metabolites with significant differences, among which 50 were significantly up-regulated and 55 were significantly down-regulated. In the positive ion mode, the main differential metabolites are flupirtine, eicosapentanoic acid, hydrocortisone succinate, compound III(S), (S)-ACPA, 3-dehydro-6-deoxoteasterone, acetyl-L-leucyl-L-leucylargininal, (2E,6Z)-N-Ethyl-2,6-nonadienamide, etc. In the negative ion mode, the main differential metabolites are (2R)-1-{[(2-Aminoethoxy)(hydroxy)phosphoryl]oxy}-3-hydroxy-2-propanyl (6Z,9Z,12Z,15Z)-6,9,12,15-octadecatetraenoate, Acetochlor ESA, his-asp., etc. (Table 2).

**Table 2:** The significant different metabolites of positive and negative ion mode.

ID	Metabolites	Model	FC	*p*-value	VIP	Up/down
Com_10079_pos (P1)	Flupirtine	+	0.48	0.03	1.58	Down
Com_10191_pos (P2)	Eicosapentanoic acid	+	2.19	0.02	1.67	Up
Com_1030_pos (P3)	Hydrocortisone succinate	+	0.08	0.04	4.06	Down
Com_10325_pos (P4)	Compound III(S)	+	0.34	0.03	1.93	Down
Com_10391_pos (P5)	(S)-ACPA	+	0.46	0.01	1.65	Down
Com_10503_pos (P6)	3-dehydro-6-deoxoteasterone	+	2.01	0.05	1.55	Up
Com_10526_pos (P7)	Acetyl-L-leucyl-L-leucylargininal	+	0.46	0.01	1.65	Down
Com_10570_pos (P8)	(2E,6Z)-N-Ethyl-2,6-nonadienamide	+	3.09	0.01	2.45	Up
Com_10875_pos (P9)	Tosedostat	+	2.90	0.04	1.95	Up
Com_10936_pos (P10)	1-[(8Z,11Z,14Z)-icosatrienoyl]-sn-glycero-3-phosphocholine	+	2.52	0.03	1.71	Up
Com_1036_neg (N1)	(2R)-1-{[(2-Aminoethoxy)(hydroxy)phosphoryl]oxy}-3-hydroxy-2-propanyl (6Z,9Z,12Z,15Z)-6,9,12,15-octadecatetraenoate	−	2.70	0.01	2.38	Up
Com_1131_neg (N2)	Acetochlor ESA	−	2.34	0.04	2.08	Up
Com_1164_neg (N3)	his-asp	−	0.23	0.01	3.47	Down
Com_1193_neg (N4)	Cicloprofen	−	0.45	0.01	1.88	Down
Com_1333_neg (N5)	Pentaerithrityl tetranitrate	−	0.09	0.05	3.95	Down
Com_1362_neg (N6)	Bolandiol dipropionate	−	0.45	0.01	1.78	Down
Com_1419_neg (N7)	Relacatib	−	5.38	0.03	4.03	Up
Com_1492_neg (N8)	(13Z,16Z)-docosadienoic acid	−	2.04	0.01	1.66	Up
Com_1625_neg (N9)	2-(3-Hydroxy-4-methoxyphenyl)-5,7-dimethoxy-3-chromanol	−	3.01	0.03	2.22	Up
Com_1650_neg (N10)	Methyl indole-3-acetate	−	0.41	0.00	1.97	Down

**Figure 10:**
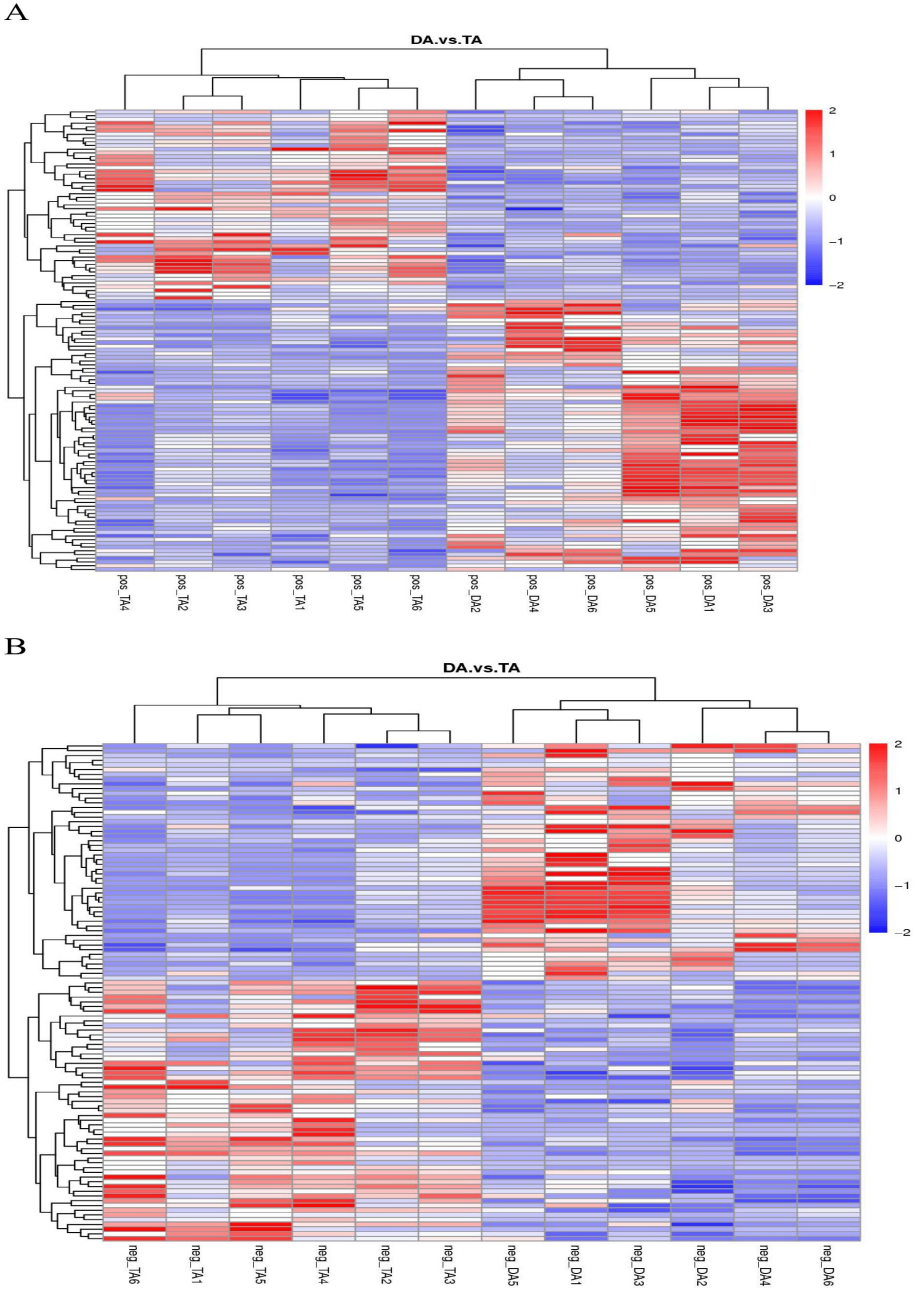
Clustering diagram of differential metabolites. **(A)** Positive ion mode. **(B)** Negative ion mode.

The cluster analysis diagram showed that the differential metabolites in the DA and TA groups are clustered, respectively, indicating that the expression patterns of metabolites in the Diannan small ear pigs and Diqing Tibetan pigs are different (Figure 10). In the positive ion mode, hydrocortisone succinic acid and acetyl-L-leucyl-L-leucylargininal were extremely significant positive correlation (*p* < 0.01); In the negative ion mode, there cicloprofen and methylindole-3-acetic acid were highly significant positive correlation (*p* < 0.01) (Tables 3, 4).

**Table 3:** The correlation of significant different metabolites of the top 10 in positive ion mode.

Metabolites	P1	P2	P3	P4	P5	P6	P7	P8	P9	P10
P1	1.000									
P2	−0.548	1.000								
P3	0.637*	−0.567	1.000							
P4	−0.019	−0.195	−0.144	1.000						
P5	0.332	−0.339	0.068	0.816*	1.000					
P6	−0.145	0.395	−0.393	−0.464	−0.497	1.000				
P7	0.581*	−0.493	0.936**	0.008	0.289	−0.510	1.000			
P8	−0.364	0.213	−0.377	−0.479	−0.628*	0.094	−0.513	1.000		
P9	−0.420	0.665*	−0.290	−0.330	−0.559	0.424	−0.420	0.489	1.000	
P10	−0.502	0.797*	−0.327	−0.371	−0.369	0.101	−0.199	0.284	0.457	1.000

^*^ indicates that the difference is significant and ^**^ indicates that the difference is extremely significant.

**Table 4:** The correlation of significant different metabolites of the top 10 in negative ion mode.

Metabolites	N1	N2	N3	N4	N5	N6	N7	N8	N9	N10
N1	1.000									
N2	0.622*	1.000								
N3	−0.535	−0.395	1.000							
N4	−0.579*	−0.712*	0.645*	1.000						
N5	−0.690*	−0.670*	0.175	0.726*	1.000					
N6	−0.553	−0.674*	0.165	0.365	0.358	1.000				
N7	0.780*	0.619*	−0.432	−0.335	−0.400	−0.420	1.000			
N8	0.505	0.278	−0.748*	−0.737*	−0.284	−0.344	0.156	1.000		
N9	0.483	0.349	−0.652*	−0.629*	−0.248	−0.180	0.384	0.713	1.000	
N10	−0.697*	−0.789*	0.453	0.875**	0.752*	0.707*	−0.357	−0.672*	−0.495	1.000

^*^ indicates that the difference is significant and ^**^ indicates that the difference is extremely significant.

**Figure 11:**
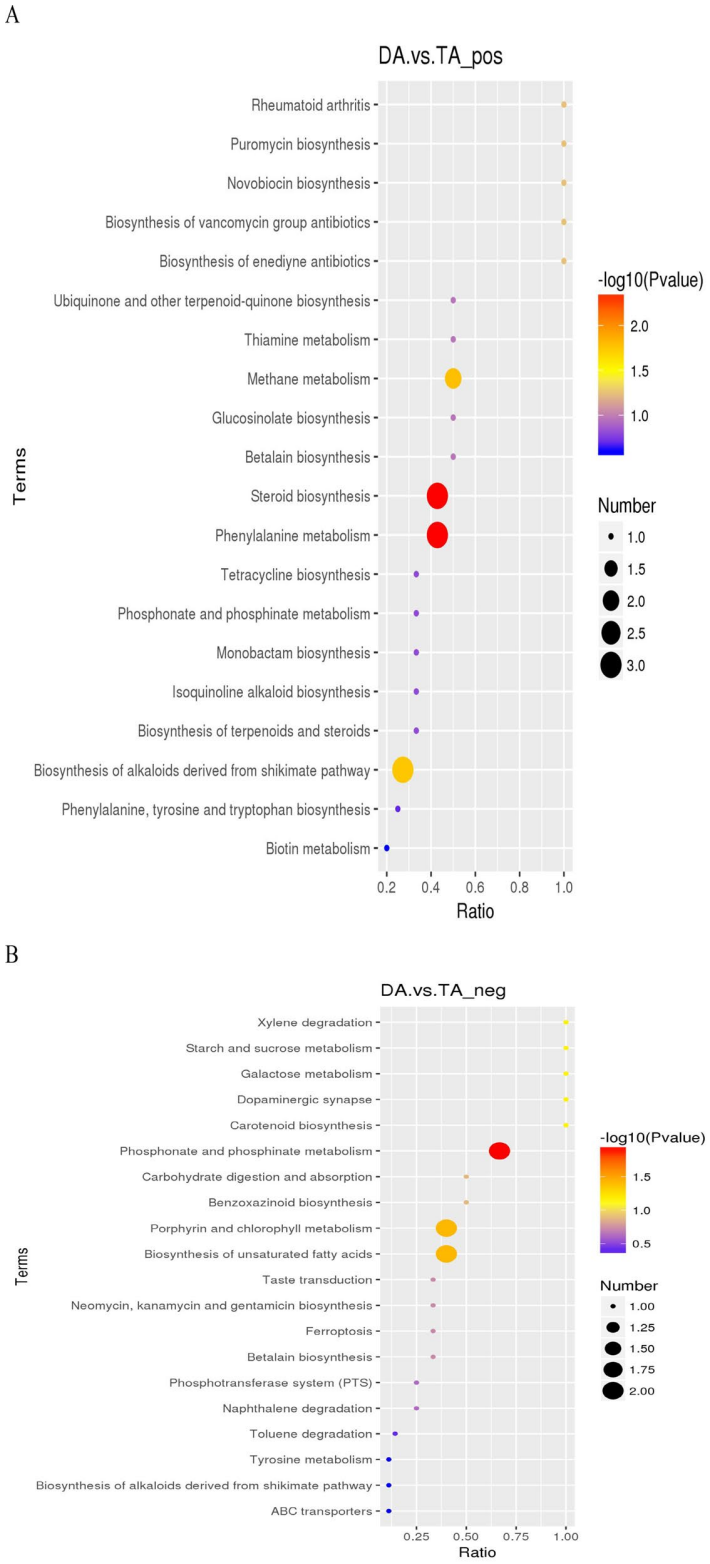
KEGG enrichment map. **(A)** Positive ion mode. **(B)** Negative ion mode.

KEGG analysis (Figure 11) showed that in the positive ion mode, the metabolic pathways with significant differences were mainly steroid biosynthesis involved by cholecalciferol, 5-dehydroepisterol and stigmasterol; phenylalanine metabolism involved by 3-phenylpropanoic acid, L-tyrosine, phedrine; methane metabolism involved by L-tyrosine, 7-oxoheptanoic acid; Biosynthesis of alkaloids derived from shikimate pathway involved by L-tyrosine, tetrahydroamine and ephdrine. In the negative ion mode, the metabolic pathways with significant differences were mainly phosphonate and phosphinate metabolism involved by rhizoctin B, rhizoctin D; Porphyrin and chlorophyll metabolism involved by stercobilin, pyrophoride a; Biosynthesis of unsaturated fatty acids involved by adrenic acid, docosahexaenoic acid.

### Analysis of correlations between different microbiota and metabolites

In the positive ion mode, hydrocortisone succinate with *Clostridium_disporicum* exhibited a significantly positive correlation (*p* < 0.05). However, hydrocortisone succinate with *Bifidobacterium_pseudocatenulatum*, *Bacteroides_stercoris*, and *Bacteroides_ovatus* exhibited a significantly negative correlation (*p* < 0.05). compound III(S) with *Porphyromonadaceae_bacterium_DJF_B175* exhibited a significantly positive correlation (*p* < 0.05). 3-dehydro-6-deoxoteasterone with *Lactobacillus_amylovorus* exhibited a significantly negative correlation (*p* < 0.05). 3-dehydro-6-deoxoteasterone with *Lactobacillus_amylovorus* exhibited a significantly negative correlation (*p* < 0.05). Acetyl-L-leucyl-L-leucylargininal with *Lactobacillus_amylovorus* exhibited a significantly positive correlation (*p* < 0.05). (2E,6Z)-N-Ethyl-2,6-nonadienamide with *Lactobacillus_amylovorus* exhibited a significantly negative correlation (*p* < 0.05). Ethyl (2R)-2-[(3S,5aS,9aR,10aS)-3-methyl-1,4-dioxodecahydropyrazino[1,2-a]indol-2(1H)-yl]-4-phenylbutanoate with *Lactobacillus_amylovorus* exhibited a significantly negative correlation (*p* < 0.05). (Carbamoylamino) (4-hydroxyphenyl) acetic acid with *Bacteroides_stercoris* exhibited a significantly positive correlation (*p* < 0.05). However (Carbamoylamino) (4-hydroxyphenyl) acetic acid with *Lactobacillus_amylovorus* exhibited a significantly negative correlation (*p* < 0.05). 1-Propyl-1H-purin-6-amine with *Lactobacillus_amylovorus* exhibited a significantly positive correlation (*p* < 0.05). 3-Phenylpropanoic acid with *Bacteroides_stercoris*, *Bacteroides_ovatus*, and *Bifidobacterium_pseudocatenulatum* exhibited a significantly positive correlation (*p* < 0.05). However, 3-Phenylpropanoic acid with *Lactobacillus_amylovorus* exhibited a significantly negative correlation (*p* < 0.05). Avasimibe with *Bacteroides_stercoris*, *Bifidobacterium_pseudocatenulatum*, *Bacteroides_vulgatus*, *Bacteroides_dorei*, and *Bacteroides_ovatus* exhibited a significantly positive correlation (*p* < 0.05).

**Figure 12:**
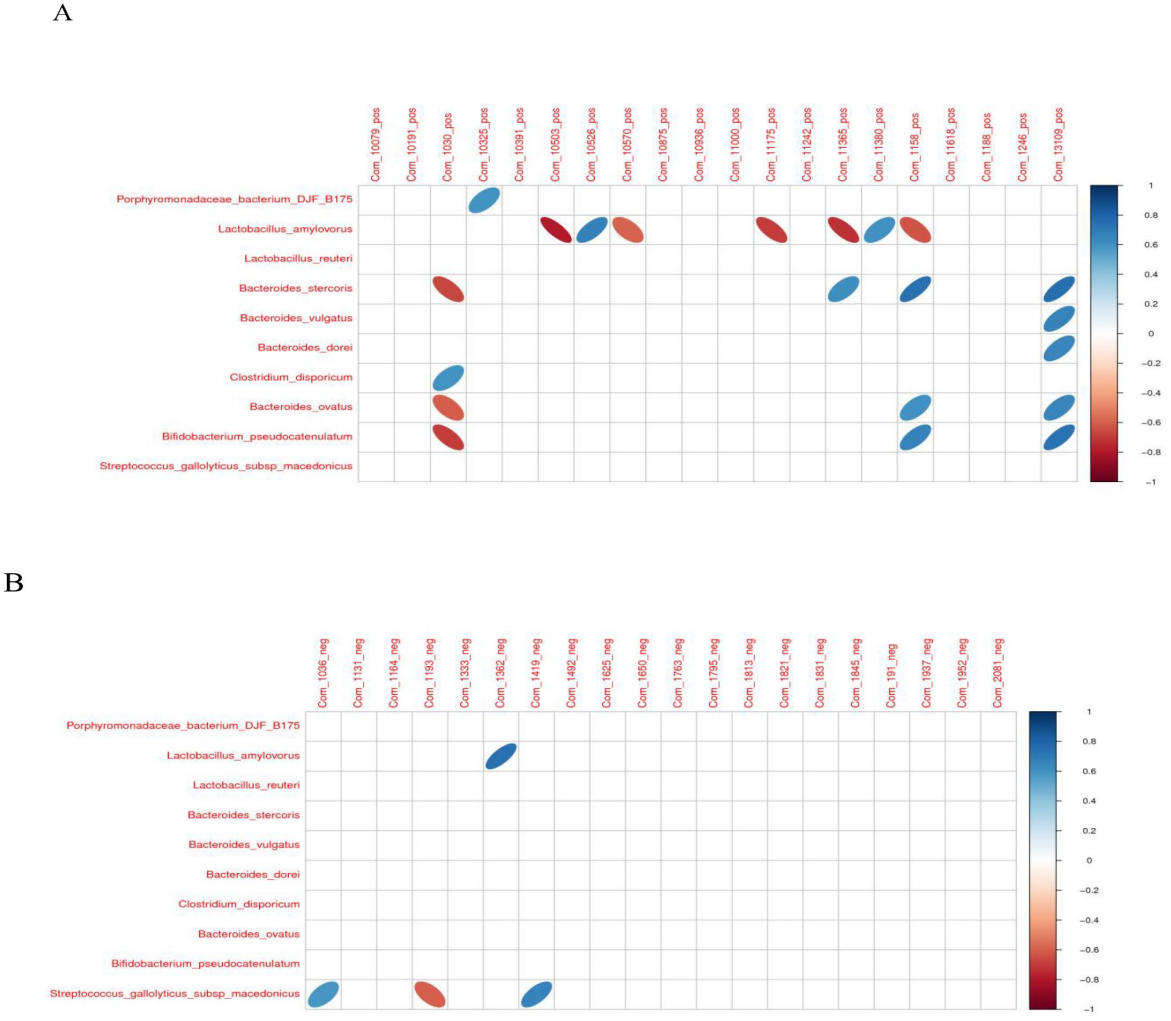
The correlation heat map of differential intestinal microbiota and metabolites. **(A)** Positive ion mode. **(B)** Negative ion mode.

In the negaative ion mode, (2R)-1-{[(2-Aminoethoxy)(hydroxy)phosphoryl]oxy}-3-hydroxy-2-propanyl (6Z,9Z,12Z,15Z)-6,9,12,15-octadecatetraenoate with *Streptococcus_gallolyticus_subsp_macedonicus* exhibited a significantly positive correlation (*p* < 0.05). Cicloprofen with *Streptococcus_gallolyticus_subsp_macedonicus* exhibited a significantly negative correlation (*p* < 0.05). Bolandiol dipropionate with *Lactobacillus_amylovorus* exhibited a significantly positive correlation (*p* < 0.05). Relacatib with *Streptococcus_gallolyticus_subsp_macedonicus* exhibited a significantly positive correlation (*p* < 0.05) (Figure 12).

## Discussion

The composition of intestinal microorganisms in different breeds of pigs is different, affecting the body’s physiological characteristics and digestive function (Wang et al., 2022). Our findings revealed that Firmicutes and Bacteroidetes are the most abundant phylum in the intestine of Diannan small ear pigs and Diqing Tibetan pigs, accounting for more than 90% of the total sequence, which is consistent with previous study results (Kim et al., 2011; Gao et al., 2019). The relative abundance of Firmicutes in Diannan small ear pigs is higher than that in Diqing Tibetan pigs. The relative abundance of Bacteroidetes is lower than that in Diqing Tibetan pigs. Firmicutes degrade fiber and cellulose (Armougom and Raoult, 2008), and Bacteroidetes help digest complex carbohydrates and ferment organic matter (Xu et al., 2019). Biddle et al. (2013) have shown that individuals with a high ratio of Firmicutes/Bacteroidetes have a higher efficiency of absorbing energy from food, thus effectively promoting the host’s energy absorption and increasing the host’s body weight. Therefore, the results of this study are consistent with the trait of more fat deposition in Diannan small ear pigs (Wang et al., 2015).

At the genus level, *Bacteroides* is related to the metabolism of proteins, various amino acids, and saturated fats (Zhang et al., 2014). *Lactobacillus* maintains intestinal and body health by inhibiting the invasion and colonization of the intestinal tract by pathogenic bacteria. At the same time, can enhance the digestive function of the intestinal tract (Valeriano et al., 2017). *Bifidobacterium* has critical physiological functions such as improving immunity, anti-aging, anti-tumor, and maintaining gastrointestinal health (Luo et al., 2018). Through studies in mice, Sokol et al. (2008) confirmed that *Clostridium flexis* has anti-inflammatory properties and plays a central role in maintaining the balance of intestinal flora. Therefore, we assume that the intestinal microbial composition of Diannan small ear pigs and Diqing Tibetan pigs may be related to their potential resistance to stress and disease.

The diversity of gut microbes can reflect the health of the host. The diversity of gut microbes are usually positively correlated with the microbiota’s stability, disease resistance and stress resistance. High diversity benefit overall health, and productivity (Yang L. et al., 2014; Yang W. et al., 2014). The environmental differences in different regions of Yunnan have formed the local pig breeding resources of Diannan small ear pigs and Diqing Tibetan pigs with excellent germplasm and unique traits (Zhang J. et al., 2015; Zhang B. et al., 2015). The results of this study show that the diversity of intestinal microorganisms in Diqing Tibetan pigs is higher than that in Diannan small ear pigs, which may be related to the fact that Diqing Tibetan pigs can adapt to the cold environment on the plateau. The interaction between genotype and environment resulted in the strong disease resistance and cold resistance of Diqing Tibetan pigs (Ge et al., 2018).

Host genetics have essential effects on specific strains of bacteria (Zhang J. et al., 2015; Zhang B. et al., 2015). This study showed significant differences in the intestinal microbial structure between Diannan small ear pig and Diqing Tibetan Pig. The relative abundance of *Lachnospiraceae*, *Actinomyces*, and *Butyricicoccus* in the feces of the Diannan small ear pigs was significantly higher than that of the Diqing Tibetan Pigs. Lachnospiraceae is a crucial beneficial bacterium which can ferment and degrade carbohydrates into SCFAs (Duncan et al., 2007; McKnite et al., 2012). *Actinomyces* are gram-positive bacteria with branching hyphae whose metabolites can block particular pathogens and modulate the body’s immunity (Forster et al., 1997). Ding et al. (2016) found that *Actinomyces* metabolites isolated from wild animal feces can decompose cellulose, enzyme activity, antibacterial activity, and anti-tumor activity. Relevant studies isolated *Butyricicoccus* from the rumen of cattle, sheep, and deer. It was found that *Butyricicoccus* can also regulate the secretion of hemicellulose degrading enzymes, degrade polysaccharides (Louis and Flint, 2009), and produce butyrate to proliferate gastric epithelial cells (Dunne et al., 2012). These microorganisms may be associated with the Diannan small ear pigs’ resilience to roughage and stress (Wang et al., 2016).

Genetic and environmental factors interact in the formation of intestinal microbial composition (Dowd et al., 2007; Kurilshikov et al., 2021). Diqing Tibetan pigs have lived in pastoral plateau areas for many years, and their diet mainly comprises of pastures (Hildebrand et al., 2013). Cellulose-related bacteria colonized in the intestine can catabolize dietary fiber in plant cell walls and produce a large amount of SCFAs for energy. Help pigs better adapt to fiber-containing diets and develop roughage tolerance. Prevotelaceae and *Ruminocus* are associated with cellulose decomposition (Yang L. et al., 2014; Yang W. et al., 2014). Our study on Diqing Tibetan pigs also detected that Prevotellaceae, *Ruminococcus*, were significantly higher than those of Diannan small ear pigs, which may be related to the herbivorous habits of the Tibetan pigs.

The metabolomics results of this study found that oleamide is the metabolite with the highest relative expression in the positive and negative ion mode of Diannan small ear pigs and Diqing Tibetan pigs. Oleamide is an endogenous fatty acid amide recognized as an endogenous sleep-inducing drug after its discovery in the cerebrospinal fluid of sleep-deprived cats (Boger et al., 1998). Oleamide promotes multiple biological functions, including sleep induction, anxiolytic effect, anti-inflammatory activity, and skeletal muscle atrophy inhibition (Cravatt et al., 1995; Ano et al., 2015; Moon et al., 2018; Kobayashi et al., 2021, 2022). In addition, hydroalcoholic lard extract containing oleamide exhibits anti-inflammatory effects on lipopolysaccharide-stimulated neutrophils and peripheral mononuclear cells, lowering the ability to create TNF-α and the activity of antioxidants and pro-inflammatory enzymes (Capó et al., 2021). As one of the primary agents for eliminating metabolites in animals, feces can potentially communicate a large quantity of information regarding the internal physiology of animals and serve as a source of chemical signals (Guan et al., 2022). Therefore, this study suggested that oleamide may be a potential marker of Diannan small ear pigs and Diqing Tibetan pigs’ feces. However, the function and mechanism of oleamide as a metabolite in Diannan small ear pigs and Diqing Tibetan pigs still need to be further studied.

The metabolomics results of this study found that the steroid biosynthesis and biosynthesis of unsaturated fatty acids of Diannan small pigs were significantly higher than those of Diqing Tibetan pigs. Steroid hormones are a class of cyclopentane polyhydrophenanthrene derivatives, mainly divided into corticosteroids and sex hormones (Bovo et al., 2016). Corticosteroids can regulate physiological processes such as glucose metabolism, water, salt metabolism, and stress response (Kapugi and Cunningham, 2019). Sex hormones are mainly related to biological sex, the development of secondary sexual characteristics, and reproductive regulation (Wonyoung and Chungsoo, 2010). The regular operation of physiological functions requires hormone metabolism in the body to maintain a stable balance and be appropriately regulated. When the body is under stress, glucocorticoids in the blood increase to regulate the metabolism of sugar, water and salt, thereby enhancing immunity (Kuo et al., 2015). Glucocorticoids can induce adipocytes to differentiate and produce free or non-esterified fatty acids (Macfarlane et al., 2008); After persistent glucocorticoid release, animals develop metabolic problems such as hyperglycemia and obesity (Slavin et al., 1994). Glucocorticoids contribute to lipid metabolism and influence the expression of hormone-sensitive lipase and fat triglyceride lipase, according to the research of Vllena (Villena et al., 2004). Unsaturated fatty acids affect lipid metabolism in animal tissues. Bessa et al. discovered that during the hydrogenation of dietary polyunsaturated fatty acids by rumen microbes of lambs, some of the intermediate products and unsaturated fatty acids produced might be deposited directly into the muscle influencing the nutritional value of the muscle (Bessa et al., 2007). The pork quality of Diannan small ear pigs is outstanding, with an unsaturated fatty acid content of about 53.22%, an intramuscular fat content of about 8.5%, and a low lean meat rate of about 35%, which is consistent with the high expression of related metabolic pathways.

In addition, the phosphonate and phosphinate metabolism and phenylalanine metabolism pathways of Diqing Tibetan pigs were significantly higher than those of Diannan small ear pigs. Aminophosphonate compounds have good antibacterial activity, and studies have found that aminophosphonate compounds can inhibit harmful *Escherichia coli* to a certain extent (Elsherbiny et al., 2020). Phenylalanine is an aromatic non-polar alpha amino acid, which can be directly converted into tyrosine to participate in protein synthesis, and it also plays a vital role in maintaining body health (MacDonald et al., 2020). These two metabolic pathways are consistent with the germplasm characteristics of Diqing Tibetan pigs with strong disease and stress resistance. In conclusion, according to the study results, it is speculated that the differential metabolic pathways enriched by differential metabolites are related to the specific germplasm characteristics of Diannan small ear pigs and Diqing Tibetan pigs.

The link between intestinal microbiota and metabolites shows that intestinal microbiota is crucial for bodily health (Chow et al., 2010). Interestingly, our study found that *Clostridium_disporicum* was significantly positively correlated with hydrocortisone succinate. Hydrocortisone is a steroid hormone that regulates multiple metabolic and physiological processes and is involved in response to many stresses (Pruett, 2003; Timmermans et al., 2019). Prior to excretion, the hydrocortisone metabolism is often complicated (Santamaria et al., 2021). During irreversible enzymatic processes, hydrocortisone is conjugated with sulfate or glucuronides to improve its solubility and permit its excretion in urine via the kidney or feces via the colon (Palme et al., 2005; Keegan, 2019). In addition, microorganisms in the gut can undertake side-chain cleavage of cortisol metabolites, which ultimately leads to the formation of fecal cortisol metabolites (Morris and Ridlon, 2017). *Clostridium_disporicum* is a saccharolytic species within Firmicutes and is associated with the decomposition of complex organic matter, but its actual actions in the intestine remain unknown (Li et al., 2021). Therefore, our study predicted that *Clostridium_disporicum* could degrade hydrocortisone to form hydrocortisone succinate in the intestine of Diqing Tibetan pigs.

## Data availability statement

The data presented in the study are deposited in the GenBank repository, accession numbers OR162609-OR164377.

## Ethics statement

The animal study was reviewed and approved by Institutional Animal Care and Use Committee of Yunnan Agricultural University. Written informed consent was obtained from the owners for the participation of their animals in this study.

## Author contributions

SZ and XG were responsible for acquisition of the data and participated in drafting the manuscript. JZ, LY, HS, MY, YH, HP, HW, HZ, and YZ participated in conception and design of the experiment and analyses of the results, performed statistical analyses, and agreed to be accountable for accuracy and integrity of the data. All authors contributed to the article and approved the submitted version.

## Funding

This work was supported by the Major Science and Technology Project of Yunnan Province (202202AE090032 and 202102AA310054), Yunnan Science and Technology Talents and Platform Program (Academician Expert Workstation) (202305AF150179), National Natural Science Foundation of China (31760645, 31260592, and 31060331), Technological Innovation Talent Program (2020FA011), and State School Cooperation (2020ZXND02).

## Conflict of interest

The authors declared that they have no conflicts of interest to this work.

## Publisher’s note

All claims expressed in this article are solely those of the authors and do not necessarily represent those of their affiliated organizations, or those of the publisher, the editors and the reviewers. Any product that may be evaluated in this article, or claim that may be made by its manufacturer, is not guaranteed or endorsed by the publisher.

## Footnotes

^1^ http://cutadapt.readthedocs.io/en/stable/

^2^ http://www.drive5.com/uparse/
