# Supplementary material for: Comparison of the gut microbiota and metabolites between Diannan small ear pigs and Diqing Tibetan pigs
Source: Front Microbiol. 2023 Jul 6;14:1197981. doi: 10.3389/fmicb.2023.1197981 (PMC10359432; doi:10.3389/fmicb.2023.1197981)
Supplement: Supplementary file 1 [file Table_1.pdf]

Supplementary Table S1 Composition and nutrient levels of basal diets (air-dry basis) %

Items	Content
Corn	64.21
Soybean meal	16.11
Rice bran	10.00
Wheat bran	7.10
Lime stone	1.08
Premix[1]	1.00
Dicalcium Phosphate	0.32
Salt	0.18
Total	100
Nutrient levels[1)]	
DE(MJ/kg)	13.81
CP	15.17
Lys	0.63
Ca	0.55
P	0.53

[1]Provided per kg of diet: 0.6 mg of I; 0.01 mg of Se; 100 mg of Zn;100 mg of Fe; 6.6 mg of Cu; 30 mg of Mn; 6,608 IU of vitamin A; 1,652 IU of vitamin D3; 27.5 IU of vitamin E; 4.4 mg of vitamin K; 6.6 mg of riboflavin; 39.6 mg of niacin; 26.4 mg of pantothenic acid; 33 µg of vitamin B12; 0.8 mg of pyridoxine; 1.1 mg of folic acid; 0.22 mg of biotin; 583 mg of choline; 0.66 mg of thiamine.

[2]ME was a calculated value, while the others were measured values.

Supplementary Table S2 The major metabolites of top 10 relative expressions in Diannan small ear pig feces of positive mode

Name_des	Model	DA
Oleamide	+	709221460.5
3-[2-[(E)-[3-(2-carboxyethyl)-5-[(4-ethyl-3-methyl-5-oxo-pyrrolidin-2-yl)methyl]-4-methyl-pyrrol-2-ylidene]methyl] 4--5-[(3-ethyl-4-methyl-5-oxo-pyrrolidin-2-yl)methyl]-4-methyl-1H-pyrrol-3-yl]propanoic acid	+	188145728.7
Roxatidine	+	119104144.7
3-(2-Oxo-2,3-dihydro-1,3-benzoxazol-3-yl)propanoic acid	+	98003906.47
Hexadecanamide	+	86712748.31
N,N-Dimethylsphingosine	+	84680155.67
(+)-Aphidicolin	+	79509370.55
13-Hydroxyoctadecanoic acid	+	77467362.81
7-Acetoxy-2-methylisoflavone	+	76612786.74
Acedoben	+	67644487.65

Supplementary Table S3 The major metabolites of top 10 relative expressions in Diqing Tibetan pig feces of positive mode

Name_des	Model	TA
Oleamide	+	720453640.8
N,N-Dimethylsphingosine	+	97262852.44
7-Acetoxy-2-methylisoflavone	+	96671135.8
Hexadecanamide	+	91333274.35
(+)-Aphidicolin	+	88930918.08
Nonate	+	78781008.86
[2-[(E)-[3-(2-carboxyethyl)-5-[(4-ethyl-3-methyl-5-oxo-pyrrolidin-2-yl)methyl]-4-methyl-pyrrol-2-ylidene]methyl]-5-[(3-ethyl-4-methyl-5-oxo-pyrrolidin-2-yl)methyl]-4-methyl-1H-pyrrol-3-yl]propanoic acid	+	76411695.39
Roxatidine	+	75406330.14
13-Hydroxyoctadecanoic acid	+	68399796.79
L-Norleucine	+	48763724.07

Supplementary Table S4 The major metabolites of top 10 relative expressions in Diannan small ear pig feces of negative mode

Name_des	Model	DA
Pentadecanoic acid	-	709221459.5
(+)-CP 55,940	-	188145728.7
Oleic acid	-	119104144.7
Myristic acid	-	98003906.47
.alpha.-Apocholic acid	-	86712748.31
(+/-)-CP 55,940	-	84680155.67
10-hydroxystearic acid	-	79509370.55
Linoleic acid	-	77467362.81
Hydrocinnamic acid	-	76612786.74
Saccharin	-	67644487.65

Supplementary Table S5 The major metabolites of top 10 relative expressions in Diqing Tibetan pig feces of negative mode

Name_des	Model	TA
Pentadecanoic acid	-	720453640.3
(+)-CP 55,940	-	97262852.44
.alpha.-Apocholic acid	-	96671135.8
Oleic acid	-	91333274.35
Myristic acid	-	88930918.08
Hydrocinnamic acid	-	78781008.86
10-hydroxystearic acid	-	76411695.39
Deoxycholic acid	-	75406330.14
Linoleic acid	-	68399796.79
(+/-)-CP 55,940	-	48763724.07
